# SOLiD-SAGE of Endophyte-Infected Red Fescue Reveals Numerous Effects on Host Transcriptome and an Abundance of Highly Expressed Fungal Secreted Proteins

**DOI:** 10.1371/journal.pone.0053214

**Published:** 2012-12-28

**Authors:** Karen V. Ambrose, Faith C. Belanger

**Affiliations:** Department of Plant Biology and Pathology, Rutgers University, New Brunswick, New Jersey, United States of America; Wadsworth Center, United States of America

## Abstract

One of the most important plant-fungal symbiotic relationships is that of cool season grasses with endophytic fungi of the genera *Epichloë* and *Neotyphodium.* These associations often confer benefits, such as resistance to herbivores and improved drought tolerance, to the hosts. One benefit that appears to be unique to fine fescue grasses is disease resistance. As a first step towards understanding the basis of the endophyte-mediated disease resistance in *Festuca rubra* we carried out a SOLiD-SAGE quantitative transcriptome comparison of endophyte-free and *Epichloë festucae*-infected *F. rubra*. Over 200 plant genes involved in a wide variety of physiological processes were statistically significantly differentially expressed between the two samples. Many of the endophyte expressed genes were surprisingly abundant, with the most abundant fungal tag representing over 10% of the fungal mapped tags. Many of the abundant fungal tags were for secreted proteins. The second most abundantly expressed fungal gene was for a secreted antifungal protein and is of particular interest regarding the endophyte-mediated disease resistance. Similar genes in *Penicillium* and *Aspergillus* spp. have been demonstrated to have antifungal activity. Of the 10 epichloae whole genome sequences available, only one isolate of *E. festucae* and *Neotyphodium gansuense* var *inebrians* have an antifungal protein gene. The uniqueness of this gene in *E. festucae* from *F. rubra*, its transcript abundance, and the secreted nature of the protein, all suggest it may be involved in the disease resistance conferred to the host, which is a unique feature of the fine fescue–endophyte symbiosis.

## Introduction

Most plants have symbiotic relationships with fungi [1] and one of the most important symbiotic relationships is that of cool season grasses with endophytic fungi of the genera *Epichloë* and *Neotyphodium* (collectively epichloae*)*. The endophyte-grass interaction is of commercial interest and is also a promising model for studies aimed at understanding symbiotic associations in general. It is well established that the *Epichloë* and *Neotyphodium* fungal endophytes of grasses confer numerous benefits to their hosts [1], [2], [3]. One of these benefits is reduced herbivory by insects and animals due to the production of toxic alkaloids. For many cultivated grasses, infection with fungal endophytes is desirable because of the dramatic insect resistance they confer. The association of the fungal alkaloids with reduced herbivory is a well-established aspect of the grass–endophyte symbiosis and the biosynthesis of the alkaloids is a topic of considerable research [3], [4]. Additionally, endophyte-infected grasses seem to inhibit other grassland species’ biomass when grown together, suggesting an allelopathic effect of these grasses [5].

The benefits to the grass hosts of fungal endophyte infection are well-established and some of the molecular details of the interaction between the two species are known. Endophyte production of reactive oxygen species (ROS) has been shown to be critical for maintaining a normal mutualistic interaction between the host and the fungus. Three fungal genes required for ROS production, *NoxA*, *NoxR*, and *RacA*, as well as a stress-activated MAP kinase (*SakA*) have been identified as critical for normal fungal growth *in planta* [6], [7], [8], [9]. In addition to the presence of fungal produced alkaloids, the concentrations of numerous other metabolites are altered in endophyte-infected plants. Nitrogenous compounds, such as total N, nitrate, total proteins, and free amino acids were reduced in endophyte-infected plants, whereas carbohydrates were increased [10], [11]. In some grass-endophyte symbiotic systems the endophyte was found to utilize plant produced asparagine and glutamine as precursors in the synthesis of lolines, and the levels of lolines synthesized by the endophyte were regulated by levels of the amino acids provided by the plant [12].

Endophyte infection is generally asymptomatic with no apparent reaction by the host, such as a hypersensitive response as seen in infections by pathogenic fungi [13]. The endophytic fungal hyphae ramify within the intercellular spaces of the aerial plant parts, in particular the leaf sheaths [14]. They grow through the host apoplastic spaces by a novel intercalary hyphal extension process, rather than exclusively by hyphal tip extension [15]. They do not invade the plant cells and must therefore obtain all their carbon and nitrogen compounds from the apoplastic space. However, the host must in some way sense the presence of the endophyte since previous studies using suppression subtractive hybridization and cross-species microarray hybridization have shown that presence of the endophyte *Neotyphodium coenophialum* results in changes in host gene expression in tall fescue (*Festuca arundinacea*, syn. *Lolium arundinaceum*) [16], [17]. Several studies indicate endophyte infection can result in increased plant vigor and confer tolerance to abiotic stress, unrelated to the reduction in herbivory [2]. The physiological mechanisms that produce these effects are not understood, but it seems likely some changes in host gene expression may be a factor.

Another well-established effect of some grass-endophyte symbioses is disease resistance, but this effect may be unique to endophyte-infected fine fescues since resistance to fungal pathogens is not an established effect of epichloae endophyte infection of other grass species [1]. The term “fine fescue” refers to several *Festuca* spp., which are characterized by their fine, tough, and bristle-like leaves [18]. Some fine fescue species are important for use as low maintenance turfgrasses [18]. Individuals of these species are often naturally infected with the fungal endophyte *Epichloë festucae* [19], [20]. Many current fine fescue cultivars are infected with endophytes and endophyte infection is generally desirable because of the insect and disease resistance conferred on the host grass. However, the fine fescue–fungal endophyte relationship has not been as well studied as that of other host grasses, such as perennial ryegrass (*Lolium perenne*) or tall fescue. In field evaluations, endophyte-infected fine fescues exhibited enhanced resistance to the fungal diseases dollar spot and red thread caused by *Sclerotinia homoeocarpa* and *Laetisaria fuciformis*, respectively [21], [22], but there is no information as to the mechanism of the observed disease resistance seen in this symbiotic association. The endophyte-mediated disease resistance is agronomically important, since it reduces the fungicide requirements for these low maintenance grasses.

New high-throughput sequencing systems have revolutionized genome sequencing as well as expression analysis [23], [24]. Now it is possible to quantitatively compare transcript abundance among many samples at relatively low cost. Such new approaches can considerably expand the information generated from previous approaches regarding the effect of endophyte infection on host gene expression. For example, RNAseq analysis was previously used to compare host and endophyte transcriptomes of *L. perenne* infected with wild-type or a MAP kinase (*sakA*) mutant *E. festucae* endophyte [9]. Their analysis revealed major changes in both the host and fungal transcriptomes in the plant infected with the mutant endophyte relative to infection with the wild type endophyte. In the mutant infection the up-regulated fungal and plant genes included many hydrolytic enzymes and pathogen defense genes, respectively, indicative of a pathogenic interaction rather than the wild-type mutualistic interaction.

Here we used SOLiD-SAGE, a high-throughput adaptation of serial analysis of gene expression (SAGE) [25] using Life Technologies’ Sequencing by Oligonucleotide Ligation and Detection (SOLiD) platform, to quantitatively compare transcript abundance in endophyte-free and endophyte-infected strong creeping red fescue (*Festuca rubra* L. subsp. *rubra*). This analysis revealed hundreds of plant genes involved in many different physiological processes whose expression levels were affected by the presence of the fungal endophyte. The analysis also revealed that a large proportion of the highly expressed fungal endophyte transcriptome is comprised of transcripts for secreted proteins, for many of which the functions are not known. One of these abundant transcripts encodes an antifungal protein that appears to be unique to epichloae endophytes infecting *F. rubra* and *Achnatherum inebrians* (drunken horse grass). It is a candidate for involvement in the observed endophyte-mediated disease resistance in *F. rubra*.

## Results

### 454 Transcriptome Sequencing

The plant materials used in the study were a strong creeping red fescue not infected with *E. festucae*, designated S1139E-, and the same plant genotype inoculated with the *E. festucae* Rose City strain, designated S1139RC [26]. The Rose City isolate was obtained from an endophyte-infected strong creeping red fescue plant. The Rose City isolate of *E. festucae* has been demonstrated to confer insect resistance and fungal pathogen resistance to its host [21], [22], [27].

The characteristics of the 454 transcriptome sequences are summarized in Table 1. We obtained over 200,000 total sequences with an overall average length of 307 bp. The S1139RC 454 sequences, most of which represent plant transcripts, were assembled into 68,817 plant unigenes (plant contigs plus plant singletons). The diploid monocot rice has 41,000 genes [28]. *F. rubra* is a hexaploid [29], so a crude estimate of the gene content is 123,000 genes. The 454 sequences therefore are estimated to cover 56% of the total *F. rubra* gene content.

**Table 1:** Characteristics of the 454 sequences.

	*Festuca rubra,* endophyte-infected (S1139RC)	*Epichloë festucae* (from culture)
**Total raw reads (n)**	122,041	79,654
Mean length of raw reads (nt)	309	305
Total nucleotides of raw reads (nt)	37,717626	24,339,130
**Post trim total reads (n)**	118,862	77,254
Post trim mean length of reads (nt)	316	314
Post trim total nucleotides (nt)	37,592,208	24,245,046
**Reads assembled into contigs (n)**	67,553	54,638
Mean length of assembled reads (nt)	287	321
**Total contigs (n)**	20,501	13,381
Contig N50	426	544
Mean length of contigs (nt)	382	470
Plant origin contigs (n)	19,496	NA
Fungal endophyte origin contigs (n)	1,005	NA
**Total singletons (n)**	51,309	22,616
Mean length of singletons (nt)	278	277
Plant origin singletons (n)	49,321	NA
Fungal endophyte origin singletons (n)	1,988	NA

### Differentially Expressed Host Plant Genes Identified by SOLiD-SAGE Sequencing

SAGE (serial analysis of gene expression) and the improved method SuperSAGE [30], [31], [32] have been used in numerous studies for transcriptome analysis. SuperSAGE generates 27 bp cDNA sequences, and in SAGE terminology the sequences are called “tags”. We used SuperSAGE in combination with the massive sequencing capability of the SOLiD sequencing system. SOLiD-SAGE generates 27 bp tags from the most 3′ *Nla*III restriction site (recognition site: 5′-CATG-3′) in each cDNA [25], [33]. The 4 base *Nla*III restriction site is expected to be frequent and to occur on average every 256 bp (4^4^ = 256), but if a transcript does not have an *Nla*III site it will not be represented among the tags. The number of times a particular tag is sequenced is directly related to the number of transcripts in the sample (one tag = one transcript). Relative transcript levels can therefore be determined. Triplicate biological samples for each of the plant genotypes were prepared. Triplicate biological samples are critical for evaluation of statistical significance of differential gene expression. The characteristics of the SAGE tags are summarized in Table 2. We obtained between 5 and 10 million total tags per sample. The total tags were filtered to remove any tags that did not contain the *Nla*III cleavage site (CATG) at the 5′ end. Such tags must have originated from random ligation of adaptors to the cDNA and cannot be used in a quantitative assessment of transcript abundance. Those tags containing the *Nla*III site were further filtered to remove any that had greater than 10 As at the 3′ end. The SAGE tags are generated from the most 3′ *Nla*III site and are 27 bp long. If the *Nla*III site is close to the end of the transcript, then some of the resulting tag sequence will contain part of the poly(A)^+^ region of the cDNA. Such tags will not map to the reference dataset. Since it is impossible to distinguish between As that are truly part of the transcript sequence versus As originating from poly(A)^ +^, an arbitrary number of 10 was chosen.

**Table 2:** Characteristics of the SOLiD-SAGE tags.

	Total tags	Tags with *Nla*III site	Tags after removal of those with >10 As	Plant mapped tags (% of potential mappable)	Fungal mapped tags (% of potential mappable)
S1139E-					
Replicate 1	10,266,193	7,208,225	7,194,891	4,346,031 (60%)	NA
Replicate 2	8,048,623	5,629,653	5,613,295	3,303,965 (59%)	NA
Replicate 3	6,015,959	4,019,984	4,012,492	2,269,416 (56%)	NA
S1139RC					
Replicate 1	5,415,607	3,044,831	3,041,847	1,137,472 (37%)	43,199 (1.4%)
Replicate 2	6,165,530	4,031,091	4,025,153	2,160,008 (54%)	112,294 (2.8%)
Replicate 3	5,736,324	3,364,721	3,361,001	1,471,380 (44%)	71,927 (2.1%)

The number of tags remaining after the two filtering steps was between 3 and 7 million. These tags represent the expected number of tags that could be mapped. Tags that actually mapped to the plant reference dataset ranged from 37% to 60% of the expected number of mapped tags. The difference in the expected number of mappable tags and the actual number of mapped tags is likely due to the lack of a complete *F. rubra* reference dataset. As described above, our *F. rubra* 454 sequences were estimated to cover 56% of the plant transcriptome. Additional *Festuca* and *Lolium* EST sequences from NCBI were included in the mapping database, so the overall coverage of the plant genes could be expected to be higher than 56%, but it is likely that some plant genes were not represented in the mapping database. Even with this limitation to the SOLiD-SAGE approach with a nonmodel system, millions of tags could be mapped and plant genes with differential expression could be identified.

The same tag sequence was the most abundant tag in all 6 libraries and was a match to a 454 sequence that was identified through a BlastX search as a chlorophyll a/b binding protein. For comparison of expression levels between S1139E- and S1139RC the number of tags in each biological replicate was normalized to the total number of tags per million mapped tags (TMM) in that replicate. There was no significant difference in expression level of the chlorophyll a/b binding protein between the two samples. The tag for the chlorophyll a/b binding protein represented a mean of 12,282 and 11,922 TMM of the mapped tags in S1139E- and S1139RC, respectively. Other abundant tags in all the libraries that were not significantly different between the samples were identified as originating from transcripts for metallothionein and ribulose-1,5-bisphosphate carboxylase/oxygenase small subunit protein. These three abundant plant transcripts have all been reported as abundant in many plant EST libraries and previous SAGE analyses [34], [35], [36], [37], [38] indicating that the SOLiD-SAGE libraries are a good reflection of plant transcript abundance in our samples.

The plant SAGE tags were searched for those having statistically significant (*P*<0.05) differential expression levels between S1139E- and S1139RC, and 209 such tags were identified. Most (182) of the differentially expressed genes could be assigned a protein identification and these genes fell into 31 gene ontology (GO) categories. Twenty-seven of the differentially expressed tags originated from transcripts for as yet uncharacterized proteins. A summary of differentially expressed tags by gene ontology category is presented in Table 3. Additional information on all the differentially expressed tags is presented in Table S1. Some of the differentially expressed SAGE tags were SNPs of each other and could be mapped to different 454 sequences that encoded the same protein. Such tags represent alleles or alloalleles of each other. The genes showing differential expression ranged from abundantly expressed (lipid transfer proteins) to those with relatively low expression levels (stress induced hydrophobic proteins). The fold changes of the differentially expressed genes ranged from +7.4 to –7.2.

**Table 3:** Gene ontology (GO) categorization of the 209 differentially expressed plant genes found by SOLiD-SAGE.

	GO Term	Up-regulated genes		Down-regulated genes	
		Sequences (n)	%	Sequences (n)	%
1	Autophagy	4	6	0	0
2	Carbohydrate metabolic process	0	0	2	1
3	Cell redox homeostasis	0	0	4	3
4	Cellular carbohydrate metabolic process	0	0	1	1
5	Cellular component organization	3	4	3	2
6	Cellular nitrogen compound metabolic process	2	3	2	1
7	DNA modification	2	3	0	0
8	DNA replication	0	0	1	1
9	Generation of precursor metabolites and energy	1	1	5	4
10	Lipid metabolic process	0	0	7	5
11	Meiosis	1	1	0	0
12	Metabolic process	3	4	2	1
13	Organ senescence	2	3	0	0
14	Oxidation reduction process	1	1	1	1
15	Photosynthesis	9	13	1	1
16	Protein metabolic process	2	3	0	0
17	Protein modification process	4	6	6	4
18	Protein targeting	3	4	3	2
19	Protein ubiquitination	1	1	1	1
20	Regulation of hydrolase activity	2	3	0	0
21	Response to abiotic stimulus	1	1	1	1
22	Response to biotic stimulus	1	1	1	1
23	Response to endogenous stimulus	0	0	1	1
24	Response to metal ion	1	1	1	1
25	Response to stress	8	11	10	7
26	RNA metabolic process	0	0	6	4
27	Signal transduction	2	3	8	6
28	Transcription, DNA-dependent	1	1	5	4
29	Translation	0	0	9	7
30	Transport	6	8	19	14
31	Biological process	3	4	19	14
32	No *blastx* match found	7	10	9	7
33	No match in NCBI database	2	3	9	7
	Total	72		137	

The SAGE tags for each differentially expressed gene can be found in Table S1.

One GO category of differentially expressed genes that stood out was that of photosynthesis, which comprised 13% of the up-regulated genes in the endophyte-infected plants. The SAGE tags for the up-regulated chlorophyll a/b binding protein and ribulose-1,5-bisphosphate carboxylase/oxygenase small subunit protein are from different members of the gene families for these proteins than the abundant SAGE tags discussed above. Numerous studies have reported increased productivity and photosynthetic rates in endophyte infected plants [13], although when under high light the photosynthetic rate in endophyte infected perennial ryegrass was reported to decrease [39]. Overall, the presence of the fungal endophyte resulted in modest changes in expression level for plant genes involved in a wide range of physiological processes.

Previous studies used suppression subtractive hybridization, and microarray analysis to identify plant genes differentially expressed in response to endophyte infection [16], [17]. There was little overlap with the previous studies and the differentially expressed plant genes identified here through SOLiD-SAGE. SOLiD-SAGE tags were identified for most of the plant genes reported in the previous two studies to be differentially expressed in the tall fescue-*N. coenophialum* symbiosis. However, only 1 gene, identified as from a costars family gene [40] and corresponding to TFF17 in the previous study [16], was found to have statistically significant differential expression (up-regulated) in S1139RC. The differences between this study and the previous studies may be due to the different species used as well as the different approaches for identification of differentially expressed genes.

### *E. festucae* Gene Expression *In Planta*

The SOLiD-SAGE tags from S1139RC also included transcripts for endophyte-expressed genes, which were identified by mapping to the fungal reference dataset. Since the whole genome sequence of *E. festucae* was included in the reference dataset, the fungal reference used for mapping could be expected to have near complete coverage of the fungal transcriptome. The fungal biomass in infected plants based on relative amounts of total DNA has been estimated to be less than 2% [41]. The number of fungal mapped tags ranged from 1.4% to 2.8% of the total potential mappable tags, so the percentage of mapped fungal tags is in the expected range. There were 6,298 unique fungal tags that were mapped. The number of genes in the *E. festucae* genome has been estimated to be 9,440 [3] so the SOLiD-SAGE captured tags for approximately 67% of the genes.

The fungal mapped tags were normalized as percent mapped tags in each replicate. From the means of the normalized number of tags we ranked and identified, where possible, the most abundant fungal tags, which were arbitrarily designated as those that were 0.01% and above of the total fungal mapped tags. There were 191 such tags, which accounted for 58% of the total fungal mapped tags. The remaining 42% of the fungal mapped tags constituted the bulk of the total fungal genes represented by the SAGE tags, but for many of these there was only a single tag. Identification of the *in planta* abundant fungal transcripts can reveal the metabolic processes to which the fungus is devoting its energy in the symbiotic interaction. The top 20 fungal tags are summarized in Table 4 and the complete list is presented in Table S1.

**Table 4:** The 20 most abundant *E. festucae* transcripts found in the endophyte-infected plant S1139RC.

	Mapped match	Gene identification	Mean % mapped tags
1	SRR493691.19548	NC12 (epichloae specific)	10.12
2	SRR493691.12929	Secreted, Antifungal protein (small, cysteine-rich)	6.34
3	SRR493690.59639	Secreted, Unknown function (small, cysteine-rich)	4.60
4	SRR493691.55163	Secreted, Subtilisin-like protease	2.60
5	SRR493691.16186	Unknown function (epichloae specific)	2.58
6	SRR493691.34496	Secreted, Unknown function (epichloae specific)	1.90
7	SRR493691.15406	Secreted, NC25 (gigA) (epichloae specific)	1.45
8	SRR493691.46417	Secreted, Conidiation associated	1.37
9	SRR493691.19548	Antisense, NC12 (epichloae specific)	1.17
10	SRR493691.31334	Unknown function (epichloae specific)	1.14
11	SRR493691.38494	Unknown function, glucose repressible Grg1	1.04
12	SRR493690.32961	Secreted, Unknown function	1.02
13	SRR493691.32405	Secreted, Unknown function (small, cysteine-rich) (epichloae specific)	1.01
14	SRR493691.6870	Secreted, Unknown function (small, cysteine-rich) (epichloae specific)	0.97
15	AFRX01000547	Secreted, Unknown function (small, cysteine-rich)	0.87
16	SRR493691.52692	Unknown function (epichloae specific)	0.84
17	SRR493691.35651	Secreted, Unknown function (epichloae specific)	0.76
18	SRR493691.71157	Mismatched base pair and cruciform DNA recognition protein	0.62
19	SRR493691.48167	Secreted, Unknown function (small, cysteine-rich) (epichloae specific)	0.62
20	SRR493691.69494	Secreted, Unknown function, RNase domain	0.61

The most abundant endophyte transcript expressed in the endophyte-infected plant was for a protein of unknown function. A similar transcript was reported in the *N. coenophialum*/tall fescue symbiosis and was designated NC12 [16]. The corresponding gene appears to be unique to the epichloae, since similar genes are apparently not present in any other genera for which sequence information is available. This transcript was present at exceptionally high levels in the endophyte-infected strong creeping red fescue, over 10% of the total fungal mapped SAGE tags. In contrast, the most abundant plant tag, the chlorophyll a/b binding protein, was about 1% of the mapped plant tags. The N-terminus of the encoded protein is not yet known, so the predicted cellular location of the protein cannot be determined.

The second most abundant fungal transcript, 6.34% of the fungal mapped tags, is similar to genes for secreted antifungal proteins from *Penicillium* and *Aspergillus*. Some of these proteins have been characterized and were shown to have antifungal activity against different target organisms as well as having different modes of action [42]. Some of these antifungal proteins were found to have activity against plant pathogens, and their use in plant protection has been proposed [42]. Such genes have not previously been recognized in the fungal endophytes of grasses, perhaps because they are not present in most of the epichloae for which genome sequence information is available (discussed more below).

The fourth most abundant tag was for a secreted subtilisin-like protease, which is abundantly expressed in another system, *Epichloë poae* infected *Poa secunda* subsp. *juncifolia* (synonym *Poa ampla* Merr.) [43], [44]. Another abundant transcript was for a secreted protein that was previously found, along with NC12 described above, in the *N. coenophialum*/tall fescue symbiosis and was designated NC25 [16]. NC25, also designated *gigA*, encodes a secreted protein that is cleaved post-translationally to generate multiple cyclic oligopeptides, although the function of the oligopeptides is not yet known [45].

Overall, one striking feature of the abundantly expressed fungal transcripts is that several transcripts are exceptionally abundant and a few fungal transcripts account for a large percentage of the total fungal tags; the top 5 fungal transcripts constituted over 26% of the total mapped tags. Twenty-six of the 191 most abundant fungal tags are for proteins that are apparently epichloae specific. Fifty-nine of the most abundant tags are for secreted proteins, most of which are of unknown function. Nineteen of the 59 secreted proteins, including the antifungal protein discussed above, can be characterized as small (less than 210 amino acids) cysteine-rich proteins. Small secreted cysteine-rich proteins are involved in the interactions of fungal pathogens with their hosts, including some Avr proteins [46], [47], [48], and may also have a role in the endophyte-host symbiosis.

One of the secreted small cysteine-rich proteins (0.1%) had high similarity to the virally-encoded antifungal killer protein 4 (KP4) secreted by *Ustilago maydis*. KP4 shows antifungal activity by primarily disrupting target calcium uptake to stunt growth. Inter-kingdom horizontal gene transfer has been suggested for the presence of the protein in virus, moss and fungi [49]. KP4, along with the antifungal protein discussed above, may be a candidate gene for *Epichloë*-mediated disease resistance, although KP4-like genes are present in all epichloae genomes sequenced thus far (http://csbio-l.csr.uky.edu).

Other fungal genes that may play a role in the symbiotic association with the host were also among the highly expressed genes. A gene annotated as a salicylate hydroxylase was 0.02% of the mapped tags. Salicylate hydroxylase converts salicylate, which is required for induction of systemic acquired resistance in plants, to catechol, which is not effective [50]. Expression of an *E. festucae* salicylate hydroxylase gene could be a mechanism of suppressing the host’s defense response. However, apparently no fungal salicylate hydroxylase genes have been functionally characterized, although similar genes are present in many fungal genomes. A *Fusarium* sp. was identified that could use salicylate as its sole carbon source and salicylate hydroxylase activity was detected [51], so it is possible that the endophyte salicylate hydroxylase gene is correctly annotated.

Two SAGE tags for secreted LysM domain proteins constituted 0.11 and 0.03% of the fungal mapped tags. Secreted LysM domain effector proteins from the plant pathogens *Cladosporium fulvum*, *Mycosphaerella graminicola*, and *Magnaporthe oryzae* have been shown to bind chitin and thereby suppress the chitin-triggered plant defense responses [52], [53], [54]. Such proteins may have a similar role in plant-fungal symbiotic systems. A transcript for a LysM domain protein was among the most highly expressed transcripts in the symbiotic interaction of the mycorrhizal fungus *Tuber melanosporum* with the host roots and was proposed to play a role in sequestering chitin molecules from the host defense system [55].

SAGE tags for a major facilitator superfamily (MFS) transport protein were 0.03% of the fungal mapped tags. Comparison of the corresponding protein sequence with a transporter database (http://www.membranetransport.org/) [56] identified the *E. festucae* protein as similar to tetracycline and multidrug efflux transporters. Such efflux transporters could be for transport of fungal compounds, perhaps an alkaloid, or toxic plant defense compounds out of the cell.

How the endophyte obtains its nutrients from within the host plant apoplast is not yet understood. Tags for transporters for nitrogen and carbon compounds were among the abundant tags, suggesting the transported substrates are important nutrient sources for the endophyte. Tags for an ammonium permease (0.02%) similar to the high affinity MepA permease of *Fusarium fujikuroi* [57] and 4 amino acid permeases were among the abundant SAGE tags. Ammonium and amino acids are expected to be present in the apoplast [2], so both sources of nitrogen are apparently being utilized by *E. festucae*. The *E. festucae* genome (http://www.endophyte.uky.edu/) contains 21 genes annotated as amino acid permeases, but SAGE tags were recovered for only 9; 5 of which were of low abundance (<0.005%). Based on comparison with the functionally characterized amino acid permeases of the *Saccharomyces cerevisiae* [58] the abundantly expressed *E. festucae* genes were annotated as a proline permease similar to the proline specific Put4p permease (0.03%), 2 different arginine permeases (0.02% each), and an amino acid permease related to a choline transporter (0.01%).

Tags for four transporter proteins that may play a role in fungal carbon acquisition were also among the most abundant fungal tags. A malic acid/C4-dicarboxylate transporter was 0.08% of the mapped tags. The *E. festucae* malic acid/C4-dicarboxylate transporter is similar to that of *Schizosaccharomyces pombe*, which was functionally characterized and shown to be involved in uptake of malate, succinate and malonic acid [59]. Malate is known to be present in the apoplast [2], [60], [61], so the abundance of SAGE tags for a malate transporter suggests that malate may serve as a carbon source for the fungus. Malate levels were increased in endophyte-infected ryegrass and malate was proposed to be important for lipid biosynthesis in the lipid storing epichloae [10].

The predominant sugars expected to be present in the apoplast are sucrose, glucose, and fructose [2]. SAGE tags for a gene similar to a functionally characterized fructose specific transporter from *Botrytis cinerea* [62] were 0.02% of the mapped tags. A glucose/xylose transporter was also 0.02% of the fungal mapped tags and is similar to transporters characterized from *Candida intermedia* (GXS1) and *Colletotrichum graminicola* (CgHXT1) [63], [64]. The *C. intermedia* protein was shown to transport both glucose and xylose and the *C. graminicola* protein transported glucose, mannose, fructose, and xylose. The possibility that the *E. festucae* protein could transport xylose in addition to glucose is of interest since xylose is abundant in the hemicellulose component of grass cell walls [65]. A similar, yet distinct (since the best match to the *E. festucae* genome is on a different contig), transporter from *N. lolii* was functionally characterized and shown to preferentially transport mannose into the cell [66]. These authors also detected the presence of a fungal mannosidase in the infected plant and therefore hypothesized the endophyte has the capability to access cell wall carbohydrates. SAGE tags for an *E. festucae* transcript similar to the characterized mannose transporter were present, but at lower abundance (0.002%) than those for the glucose/xylose transporter (0.02%).

The possibility of *E. festucae* using xylose as a carbon source *in planta* is supported by the abundance of SAGE tags for enzymes required for utilization of xylose. A secreted ß-xylosidase (0.01%) could hydrolyse extracellular xylo-oligosaccharides to xylose [67], which could then be transported into the cell via the glucose/xylose transporter (0.02%). The cytoplasmic enzymes xylose reductase (0.02%), xylitol dehydrogenase (0.01%), and xylulose kinase (0.01%) together could act to convert xylose to xylulose-5-phosphate, which could then enter the pentose phosphate pathway [68]. *E. festucae* was able to grow in culture with xylose as the sole carbon source [69].

SAGE tags for a gene similar to the functionally characterized *Saccharomyces cerevisiae* alpha-glucoside transporter AGT1 were 0.01% of mapped tags. The yeast AGT1 transporter has high affinity for sucrose and lower affinity for maltose and maltotriose [70], [71]. Sucrose transported into the fungal cell could then be broken down to glucose and fructose by a cytoplasmic invertase (0.01%). SAGE tags for a secreted invertase were recovered at a lower level (0.002%). Apparently *in planta* the endophyte can gain access to the host synthesized sucrose both through direct uptake followed by cytoplasmic hydrolysis and through apoplastic hydrolysis, followed by uptake of the derived glucose and fructose, as was previously proposed for *E. festucae* in culture (72).

SAGE tags for another gene annotated as a maltose permease were 0.02% of the mapped tags. No fungal genes with high similarity to this *E. festucae* gene have been functionally characterized. The alpha-glucoside maltose is a disaccharide degradation product of starch, and is therefore unlikely to be present in the plant apoplast. However, other alpha-glycosides could be formed from degradation of the hemicellulose polymers present in grass cell walls and may be available to the fungus.

The sugar alcohols mannitol and d-arabitol are commonly found as abundant compounds in fungi and numerous functions for them have been proposed, although many proposed functions are not supported by experimental evidence [73]. Mannitol levels were correlated with endophyte biomass in infected ryegrass [10] and arabitol accumulated in endophyte-infected tall fescue under drought stress [74]. SAGE tags for a gene similar to a novel d-arabitol dehydrogenase involved in the formation of arabitol from d-xylulose in *Uromyces fabae* [75] were 0.08% of mapped tags. In fungi mannitol-1-phosphate 5-dehydrogenase converts d-fructose 6-phosphate to d-mannitol 1-phosphate, which is then dephosphorylated to mannitol. SAGE tags for mannitol-1-phosphate 5-dehydrogenase were 0.07% of mapped tags. The only mannitol-1-phosphate phosphatase so far characterized is from the protozoan parasite *Eimeria tenella* [76]. However, putative fungal mannitol-1-phosphate phosphatases have been identified as proteins having a haloacid dehalogenase-like domain, since other sugar phosphatases are known to have this domain [77]. SAGE tags to a gene annotated as a member of the haloacid dehalogenase superfamily and similar to the fungal putative mannitol-1-phosphate phosphatases were 0.03% of mapped tags. Mannitol dehydrogenase (0.04%) catalyses the oxidation of mannitol to fructose, which can then be utilized in general metabolism.

The generation of and protection from reactive oxygen species (ROS) are critical factors in plant-pathogen interactions [78], [79] and are also critical in the endophyte-grass symbiosis [80], [81], [82], [83]. Production of reactive oxygen species by *E. festucae* through the activity of the NADPH oxidase NoxA, is known to be an important factor in the maintenance of the mutualistic symbiosis, probably by regulating hyphal growth and branching of the fungus *in planta* [6], [80]. SAGE tags for the *E. festucae* NoxA were recovered at 0.003%.

SAGE tags for other genes relevant to ROS were among the abundant tags. From a proteomic analysis high levels of a fungal cytoplasmic superoxide dismutase were found in the *Neotyphodium lolii/Lolium perenne* symbiosis [84]. The authors proposed the enzyme was important in protecting the endophyte from reactive oxygen species. SAGE tags for a similar superoxide dismutase (0.11%) as well as a copper chaperone for superoxide dismutase (0.01%) [85] were among the abundant tags, consistent with the previous study. SAGE tags for other genes involved in protection from oxidative stress were glutaredoxin (0.15%), glutathione S-transferase (0.05%), and a secreted thioredoxin reductase (0.02%). SAGE tags for a secreted galactose oxidase, which generates hydrogen peroxide, were 0.08%. Galactose is typically present in the apoplast [2] so galactose oxidase may be a source of the endophyte ROS proposed to enhance leakage of nutrients from the host cells and to induce plant synthesis of antioxidants [81].

One of the major benefits of endophyte infection to the grass host is protection from insect and mammalian herbivory mediated by the synthesis of toxic alkaloids, which are generally found *in planta* and not in culture. There are four classes of alkaloids associated with protection from herbivory and *E. festucae* can produce all four, although no single isolate is known that produces all four [19]. The endophyte-infected plant genotype used in this study, S1139RC, was previously analyzed for three of the alkaloid classes. The ergot alkaloid ergovaline and the indole-diterpene lolitrem B were detected, but there was no peramine detected [27]. The pyrrolopyrazine alkaloid peramine, an insect feeding deterrent, is synthesized via a non-ribosomal peptide synthetase (*perA*) [86]. There was an *E. festucae* 454 sequence that was a good match to the *perA* gene, however, there was no SAGE tag corresponding to the *perA* gene. Whether the Rose City isolate of *E. festucae* can actually produce peramine is not yet known. The indole-diterpenes and the ergot alkaloid ergovaline are mammalian mycotoxins that each require 11 genes for their biosynthesis [4], [41], [87], [88], [89]. The loline alkaloids have insecticidal properties and also require 11 genes for their synthesis [4], [90]. None of the genes for these 3 classes of alkaloids were represented in either of the 454 sequence datasets or the SAGE tags, although this *E. festucae* isolate is known to produce both lolitrem and ergovaline. The expression level of the alkaloid biosynthetic genes in S1139RC must be relatively low, such that their transcripts were not captured in the sequencing described in this study.

### Antisense SAGE Tags

Antisense transcripts have been found in many organisms and are considered to have regulatory roles in gene expression [91], [92]. However, the exact mechanism of action and function of these molecules remains unclear. The directionality of SAGE tags allows the detection of antisense transcripts. Antisense SAGE tags have been reported in rice, *A. thaliana*, wheat, sugarcane, and human transcriptome studies [35], [36], [93], [94], [95], [96]. Eight antisense tags were among the 191 most abundant fungal tags (Table S1). The most abundant antisense tag (1.17% of mapped tags) was for NC12, which also was the most abundant sense tag (10.12%). Other abundant fungal antisense tags were for NC25 (0.13%), plasma membrane proteolipid (0.04%), subtilisin-like protease (0.03%), and four genes of unknown function (0.18%, 0.12%, 0.08%, 0.05%). The abundance of the fungal antisense tags relative to the corresponding sense tag ranged from 1∶9 (NC12) to 1∶87 (subtilisin-like protease). One of the antisense tags for a gene of unknown function (0.05%) did not have a matching sense tag.

**Figure 1:**
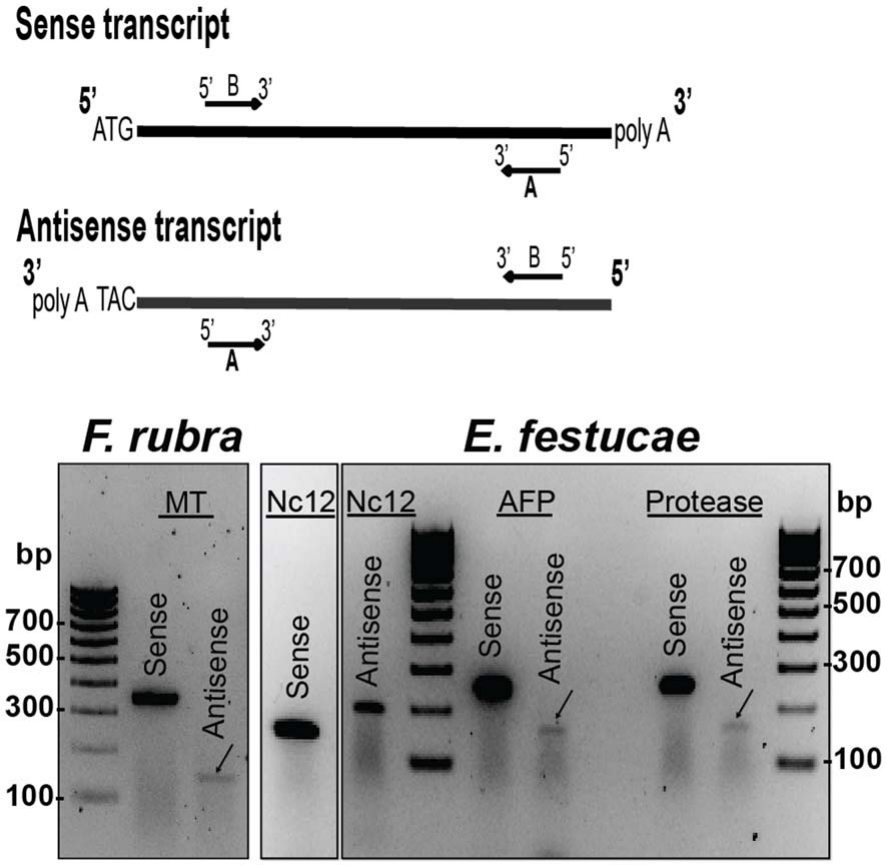
Gel analysis of *F. rubra* and *E. festucae* antisense transcripts. The diagram illustrates primer design for detection of sense and antisense transcripts. The “A” primers were used for strand specific synthesis of cDNA from the RNA sample. The “A” and “B” primers were used for cDNA amplification. cDNAs generated from gene-specific primers for the *F. rubra* metallothionein (MT) and the *E. festucae* NC12, antifungal protein (AFP), and subtilisin-like protease were used as templates for PCR amplification.

Since fungal antisense tags were identified, the plant SAGE tags were searched for the presence of antisense tags. Antisense tags were identified among the plant SAGE tags for metallothionein, chlorophyll a-b binding protein, and non-specific lipid-transfer protein. The plant antisense tags were present at low abundance, ranging from 16 (non-specific lipid transfer protein) to 85 (chlorophyll a-b binding protein) per MMT. A plant antisense tag for omega 6 fatty acid desaturase was among the differentially expressed tags, as was the sense tag for the same transcript. The abundance of the plant antisense tags relative to the corresponding sense tags ranged from 1∶140 (chlorophyll a-b binding protein) to 1∶373 (non-specific lipid-transfer protein), considerably lower than that of the fungal antisense tags discussed above. Reverse transcriptase PCR (RT-PCR) using strand-specific primers for cDNA synthesis was used to confirm the presence of antisense plant and fungal transcripts in RNA isolated from S1139RC (Fig. 1).

### The Highly Expressed *E. festucae* Antifungal Protein Gene is Not Present in Many *Epichloë* spp

The second most abundantly expressed fungal protein stood out as being of interest regarding the disease resistance conferred on the host plant by *E. festucae*. This protein has been designated as an antifungal protein and was similar to proteins from *Penicillium* and *Aspergillus*, that have been demonstrated to have antifungal properties [42]. Since the epichloae endophyte-mediated disease resistance is unique to the fine fescues infected with *E. festucae*, presumably there is some unique feature of those symbioses that is responsible for the disease resistance. Whole genome sequences are available for 10 epichloae species.: *E. festucae* E2368 (Accession ADFL02000000), *E. festucae* Fl1 (Accession AFRX01000000), *E. amarillans* E57 (Accession AFRF01000000), *E. brachyelytri* E4804 (Accession AFRB01000000), *E. glyceriae* E277 (Accession AFRG01000000), *E. typhina* E5819 (Accession AFSE01000000), *N.* gansuense E7080 (Accession AFRE01000000). Sequences for *E. elymi*, *E. typhina* E8 and *N. gansuense* var. *inebrians* are available at http://csbio-l.csr.uky.edu. One of our *E. festucae* 454 sequences with the complete coding sequence of the antifungal protein gene (Accession SRR493691.12929) was used in BlastN and TblastN searches of the epichloae genomes. Matches were found only in *E. festucae* E2368 and *N. gansuense* var. *inebrians*, suggesting the other epichloae genomes do not have a gene for the antifungal protein. Nothing is known regarding whether infection by *N. gansuense* var. *inebrians* confers disease resistance to its host grass, *Achnatherum inebrians*.

It was surprising that *E. festucae* Fl1 apparently did not have an antifungal protein gene since the gene was present in the *E. festucae* E2368 whole genome sequence and in *E. festucae* isolated from *F. rubra* in this study. *E. festucae* E2368 is a strain that resulted from the hybridization of *E. festucae* isolates from *F. rubra* and *F. gigantea* [3], [97], and thus shares genome components with *E. festucae* from *F. rubra*. *E. festucae* Fl1was isolated from another fine fescue species, hard fescue (*F. longifolia*) cultivar SR3000 [41]. This hard fescue cultivar was reported to exhibit endophyte-mediated disease resistance [21], yet its endophyte apparently does not have the antifungal gene. The fact that the gene is not found in the whole genome sequence of *E. festucae* Fl1 does not exclude its involvement in the endophyte-mediated disease resistance in *F. rubra*. The basis of disease resistance in the two symbiotic associations may be different. Whether the *E. festucae* antifungal protein is a factor in the reported disease resistance of endophyte-infected *F. rubra* will require additional studies, but based on its high level of expression it should be considered a candidate gene.

**Figure 2:**
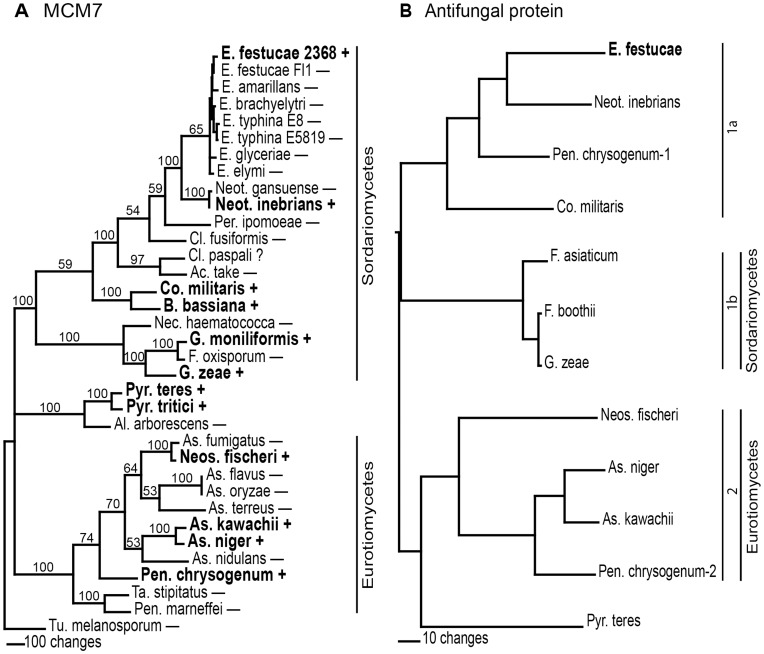
The phylogenetic relationships of the MCM7 and antifungal protein coding sequences. A. Rooted 50% majority rule maximum parsimony phylogenetic tree of the MCM7 coding sequences. The *Tu. melanosporum* sequence was designated as the outgroup for rooting the tree. The numbers at the nodes are the bootstrap percentages based on 1,000 replications. The presence (+) or absence (−) of an antifungal protein gene is indicated for each species in the tree. B. The single most parsimonious phylogenetic tree recovered from an exhaustive search of the antifungal protein coding sequences. The tree is midpoint rooted. Accession numbers of the sequences used for both trees are given in Table 5.

Nonetheless, the presence of an abundantly expressed gene, similar to antifungal protein genes from *Penicillium* and *Aspergillus,* apparently only in 2 of the epichloae genomes is interesting and raises the question of whether the gene was the result of gene gain or gene loss. Gene gain or loss can often be inferred by placing presence or absence of the gene on a species phylogeny, as well as by comparing a species phylogeny with a gene phylogeny [98]. We generated a species phylogeny based on the conserved MCM7 gene, a subunit of the hexomeric minichromosome maintenance complex (MCM), which is involved in DNA replication initiation [99]. MCM7 was previously shown to generate a robust fungal phylogeny across a wide evolutionary distance [100]. Maximum parsimony phylogenetic analysis of MCM7 sequences from fungal species within the genera that have antifungal protein genes, as well as some related species, is shown in Fig. 2A. The sources of the sequences used in the analysis are listed in Table 5. The tree was based upon 2480 total characters, of which 870 were constant, 142 variable characters were parsimony uninformative, and 1468 variable characters were parsimony informative. The sequence from the Pezizomycete *Tuber melanosporum* was designated the outgroup for rooting the tree. The relationships of the MCM7 sequences from the selected fungal species are as expected based on fungal species phylogeny [101]. The presence or absence of an antifungal protein gene similar to that found in *E. festucae* in each of the fungal species included in the MCM7 phylogenetic tree is indicated. The distribution of the antifungal protein gene was patchy, with some genera having species that both have and do not have the gene.

**Table 5:** Accession numbers of the MCM7 and antifungal protein sequences used in the phylogenetic analyses presented in Fig. 2.

Species	Accession number	
	MCM7	Antifungal protein
*Aciculosporium take* 1	AFQZ01000467	NA
*Alternaria arborescens* 1	AIIC01000090	NA
*Aspergillus flavus* 1	XM_002377869	NA
*Aspergillus fumigatus* 1	XM_750254	NA
*Aspergillus kawachii* 1	BACL01000094	GAA86026
*Aspergillus nidulans* 1	XM_658504	NA
*Aspergillus niger* 1	XM_001397760	XP_001391221
*Aspergillus oryzae* 1	XM_001826176	NA
*Aspergillus terreus* 1	XM_001213626	NA
*Beauveria bassiana* 1	ADAH01000069	ADAH01000656
*Claviceps fusiformis* 1	AFRA01000297	NA
*Claviceps paspali* 1	AFRC01000014	NA
*Cordyceps militaris* 1	AEVU01000284	EGX93824
*Epichloë amarillans* 1	AFRF01000144	NA
*Epichloë brachyelytri* 1	AFRB01001052	NA
*Epichloë elymi* 1	http://www.endophyte.uky.edu/	NA
*Epichloë festucae* 2368^1^	ADFL02000041	SRR493691.12929
*Epichloë festucae* Fl1^1^	AFRX01000012	NA
*Epichloë glyceriae* 1	AFRG01000147	NA
*Epichloë typhina* E5819^1^	AFSE01000068	NA
*Epichloë typhina* E8^1^	http://www.endophyte.uky.edu/	NA
*Fusarium asiaticum*	NA	CAR79023
*Fusarium avenaceum*	NA	CAR79018
*Fusarium boothii*	NA	CAR79010
*Fusarium cerealis*	NA	CAR79014
*Fusarium oxysporum* 1	AGNB01000011	NA
*Fusarium poae*	NA	CAR79017
*Gibberella moniliformis* 1	AAIM02000101	AAIM02007600
*Gibberella zeae* 1	AACM02000242	XP_384921
*Nectria haematococca* 1	XM_003051643	NA
*Neosartorya fischeri* 1	XM_001260497	XP_00126258
*Neotyphodium gansuense* 1	AFRE01000016	NA
*Neotyphodium gansuense* var *inebrians* 1	http://www.endophyte.uky.edu/	http://www.endophyte.uky.edu/
*Penicillium chrysogenum* 1	XM_002565874	XP_002557660
*Penicillium marneffei* 1	XM_002146315	NA
*Periglandula ipomoeae* 1	AFRD01000429	NA
*Pyrenophora teres* 1	AEEY01004277	AEEY01001915
*Pyrenophora tritici-repentis* 1	AAX101000415	XM_001934290
*Talaromyces stipitatus* 1	XM_002478600	NA
*Tuber melanosporum* 1	CABJ01000538	NA

NA indicates not applicable.

1 Species for which whole genome sequences are available.

Of the 10 epichloae for which whole genome sequences are available only one other, that of *Neotyphodium gansuense* var *inebrians*, had an antifungal protein gene. Similarly to the situation with the two sequenced *E. festucae* isolates, *N. gansuense* var. *inebrians* has an antifungal protein gene whereas the closely related but morphological distinct isolate *N. gansuense* [102] did not.

A maximum parsimony phylogenetic analysis of the mRNA sequences of the antifungal proteins revealed some unexpected relationships (Fig. 2B). The annotation of the *E. festucae* antifungal protein gene was modified from that presented with the genome sequence and is based on the 454 sequence from this study. Extensive searches of the NCBI databases have found similar genes in only two classes of fungi, the Eurotiomycetes and Sordariomycetes, with the exception of *Pyrenophora* spp., which are in the class Dothideomycetes. In order to carry out an exhaustive search, sequences from some of the species were excluded from the analysis if there was a similar sequence from a closely related species. There is a similar sequence in the *Claviceps paspali* genome that was not included in the analysis because it may be a pseudogene since annotation would require imposing a nonconsensus intron splice site in order to generate the predicted protein sequence. Since there was no appropriate sequence with which to root the tree, it was midpoint rooted. The tree from the exhaustive search was based on 297 total characters of which 62 were constant, 44 variable characters were parsimony uninformative, and 191 variable characters were parsimony informative. The antifungal protein sequences that grouped in clades 1b and 2 conform to the species phylogeny in that the Eurotiomycetes species and the Sordariomycetes species are in separate clades. However, clade 1a, which includes the *E. festucae* sequence, deviates from the species phylogeny in that it includes a *Pennicillium chrysogenum* antifungal protein sequence. *P. chrysogenum* was the only species identified that had two antifungal protein genes in its genome. Clearly, the antifungal protein gene has a complex evolutionary history. The patchy distribution of the gene among species within the same genus and the discordance of clade 1a in the gene phylogeny with the species phylogeny are suggestive of numerous instances of gene loss as well as the possibility that the *P. chrysogenum* gene in clade 1a was the result of horizontal gene transfer [98], [103].

### Expression of an Alternatively Spliced Variant of the *E. festucae* Antifungal Protein Gene

**Figure 3:**
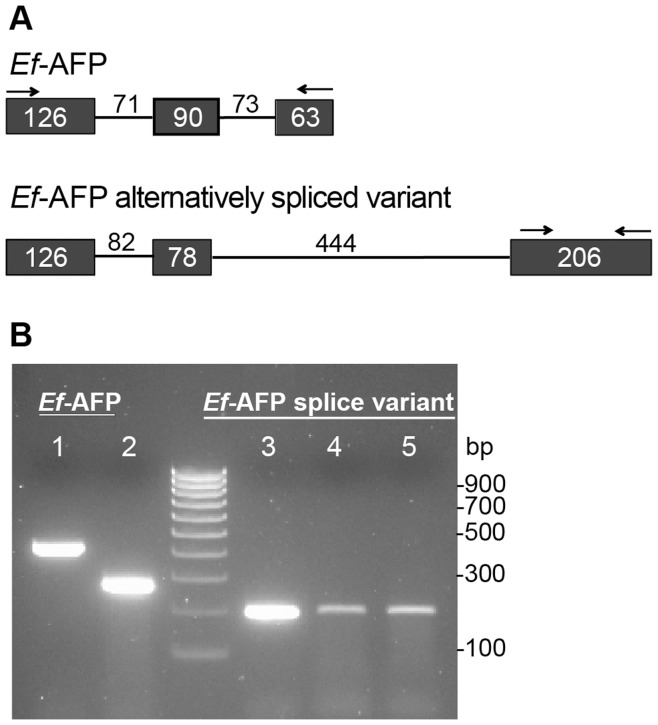
Detection of an alternatively spliced variant of *Ef-AFP*. **A.** Structural features of the *Ef-AFP* gene and the alternatively spliced variant. Exons are depicted as dark boxes, introns as lines, and sizes are given in bp. Arrows indicate positions of primers used for PCR amplification. **B.** PCR products of (1) *Ef-AFP* using *E. festucae* genomic DNA, and (2) *E. festucae*-infected plant cDNA generated from oligo(dT) as templates; (3) partial *Ef-*AFP alternatively spliced variant using *E. festucae* genomic DNA, (4) *E. festucae*-infected plant cDNA generated from a gene-specific primer (Alt *Ef-AFP* Reverse), and (5) *E. festucae*-infected plant cDNA generated from oligo(dT) as templates.

We also detected a SAGE tag that mapped to an annotated alternatively spliced variant of the *E. festucae* antifungal protein (*Ef*-AFP) in the *E. festucae* E2368 genome (http://www.endophyte.uky.edu/). The splice variant SAGE tag is 0.03% of the total *E. festucae* mapped tags while the *Ef*-AFP tag is 6.34% of the mapped tags (Table S1). The splice variant coding sequence extends downstream of the more abundant *Ef*-AFP (Figure 3A). The presence of a transcript corresponding to the SAGE tag of the alternatively spliced variant was confirmed by RT-PCR of cDNA from SR1139RC (Fig. 3B). The genome annotation predicts an intron within the amplified region. The sequence of the RT-PCR product was identical to the genome sequence, indicating there is no intron in this region. Extensive genome searches of the NCBI databases did not find any other organisms with a similar potential alternatively spliced variant in their genome.

## Discussion

Since nothing is yet known regarding the biological basis of the endophyte-mediated disease resistance in *F. rubra*, possibilities are that it may be due to fungal genes and/or fungal induction of plant genes. As a first step in determining the effect of the presence of *E. festucae* on host gene expression we carried out a quantitative transcriptome comparison between endophyte-free and endophyte-infected plants by using the SOLiD-SAGE method. The results obtained in this study highlight the usefulness of next generation sequencing technologies for non-model organisms, such as the *Festuca rubra*–*Epichloë festucae* symbiotic system. There is no genome sequence for *F. rubra* and, prior to this study, only a modest number (1,773) of EST sequences was available. For relatively low cost we generated greater than 100,000 454 EST sequences that were conservatively estimated to represent 56% of the *F. rubra* genes. Having such a homologous reference dataset greatly facilitated the ability to map the SOLiD-SAGE tags. With a more complete plant gene reference dataset, it is likely that more SAGE tags would have been mapped. In a similar study with the non-model organism *Nanomia bijuga* (Cnidaria) in which an incomplete 454 reference dataset was used, 27% of the SAGE tags were mapped [104].

In many next-generation transcriptome studies aimed at evaluating differential expression between samples, the importance of biological replication has not been recognized [105]. Statistical analysis on deep sequencing of single replicates is not a replacement for biological replication in a differential expression study. Here, we used three biological replicates of the E− and E+ samples in order to identify those SOLiD-SAGE tags whose representation in the two samples was statistically significantly different. Even with an incomplete reference dataset, over two hundred plant genes were identified that showed statistically significant differential gene expression due to the presence of the fungal endophyte. Since numerous plant genes were differentially expressed, clearly the host is sensing the presence of the endophyte in some way. The affected plant genes are involved in a wide variety of physiological processes. These results indicate that the presence of the endophyte has moderate effects on plant metabolism in many different processes. However, understanding how the observed effects on expression of individual plant genes is correlated with the overall benefits of endophyte-infection to the host will be challenging.

The effects of fungal endophyte infection on host grass metabolism are complex and influenced by the specific endophyte-host genotype interaction as well as by environmental conditions [10]. The plants used in this study were grown under growth chamber conditions in the absence of any biotic or abiotic stress. Analysis of the SAGE tags revealed hundreds of plant genes whose expression levels were moderately affected by the presence of the fungal endophyte, although there was no phenotypic difference between the endophyte-free and endophyte-infected plants. Dramatic differences in plant phenotype are often seen in field studies comparing endophyte-free and endophyte-infected plants experiencing stress, such as drought, poor soil conditions, or disease [2], [21]. Future transcriptome comparisons of endophyte-free and endophyte-infected plants experiencing stress may reveal larger differences in plant genes affected by the presence of the endophyte than those reported here. The results reported here on the fine fescue-fungal endophyte symbiosis are a first step in ultimately understanding how the differential expression of individual plant genes in response to endophyte infection results in changes in host metabolism.

The quantitative analysis of the fungal gene expression reported here revealed new information on the endophyte transcriptome *in planta*. A striking feature of the fungal SOLiD-SAGE tags was that some transcripts were present at surprisingly high levels and that many of the abundant transcripts were for secreted proteins. Fifty-nine of the 191 most abundant fungal transcripts, representing almost 31% of the abundantly expressed genes, were for secreted proteins. The size of the predicted secretomes of 9 fungal species was found to be between 5–12% of the total proteome [106], so the *E. festucae* secreted proteins are highly overrepresented in the abundant SAGE tags. The functions of most of these secreted proteins are unknown and some appear to be unique to the epichloae. Since these abundant secreted proteins are present at the interface of the two organisms, it is likely they are important in the symbiotic interaction between plant and fungus. Many of the unknown secreted proteins could be characterized as small secreted cysteine-rich proteins, which are known to be important in plant pathogen interactions [47] as well as in cell-to-cell signaling in plant development [107]. Also among the most abundant fungal tags were tags for antisense transcripts, which may play a role in regulating the expression of the even more abundant sense transcripts.

Two outstanding questions in understanding the epichloae-grass symbiotic system are, how does the fungus evade the plant defenses and what is the mechanism of nutrient acquisition by the fungus. Fungal tags for two secreted LysM domain proteins, a salicylate hydroxylase, and an MFS drug efflux transporter, an ammonium permease, 4 amino acid permeases, a malate transporter, a fructose specific transporter, a glucose/xylose transporter, a sucrose transporter and an alpha-glucoside transporter were among the most highly expressed tags and are candidates for involvement in these two critical processes. Sucrose, glucose, and fructose are known to be components of the apoplast [2] and thus likely carbon sources for the endophyte. A previous study measuring radiolabeled sugar uptake by *E. festucae* in culture concluded there were separate transporters for sucrose, glucose and fructose [72]. Here, SAGE tags for transporters for these three sugars were among the most abundant tags. A surprising finding was the abundance of SAGE tags for genes involved in xylose utilization. Xylose is abundant in the hemicellulose fraction of grass cell walls but is not known to be available as a free sugar in the apoplast. This finding supports the hypothesis of Rasmussen et al. [66] that the endophyte may be able to access some of the cell wall sugars without causing detectable damage to the cell walls.

Analysis of the plant genes whose transcript levels were affected by the presence of the fungal endophyte did not reveal any strong candidates for genes directly related to enhanced disease resistance. However, one of the fungal abundant secreted proteins is of particular interest regarding the disease resistance observed in endophyte-infected fine fescues. This protein is similar to characterized antifungal proteins from *Penicillium* and *Aspergillus* [41]. The uniqueness of this gene in *E. festucae* from *F. rubra*, its transcript abundance, and the secreted nature of the protein, all suggest it may be involved in the disease resistance conferred to the host, which is a unique feature of the fine fescue–endophyte symbiosis. The possibility that it may be involved in conferring disease resistance to the host makes this an extremely interesting protein for further research. Overall, this study has identified numerous plant and fungal genes likely to be important in the endophyte-grass interaction that are candidates for future functional characterization.

## Materials and Methods

### Plant and Fungal Materials

Strong creeping red fescue plants S1139E- and S1139RC were described previously [26]. The endophyte-infected plant S1139RC was generated by inoculating an isolated tiller of the uninfected plant S1139E- with the Rose City isolate of *E. festucae*, which was isolated from an unrelated endophyte-infected strong creeping red fescue [26]. The S1139E- and S1139RC plants thus represent endophyte-free and endophyte-infected examples of the same plant genotype. The endophyte status of the plants was confirmed microscopically prior to their use in this study. These plants can be clonally propagated and were maintained in 6-inch pots in a greenhouse.

Plants for RNA isolation for SOLiD-SAGE analysis were grown in a growth chamber set to 16 h light (400 µmol m^−2^ s^−1^) at 21C, 8 h dark at 19C, and 50–55% relative humidity. Seventy-two individual tillers of each plant type were planted in plastic 6-cell trays (one tiller per cell) and maintained in the growth chamber for 56 days. After 1 week in the growth chamber the plants were fertilized with 10-3-20 Peat Lite Plant Starter (Everris) and 30 pellets of Osmocote 14-14-14 (Scotts Miracle-Gro) were added to each cell.

*E. festucae* was isolated from the endophyte-infected S1139RC plant by plating surface-sterilized leaf sheath tissue on potato dextrose agar (Difco Laboratories, Detroit, MI).

### RNA and DNA Isolation

For RNA for the 454 pyrosequencing, leaf sheath tissue of S1139RC grown in the greenhouse was used. For RNA for the SOLiD-SAGE library preparation, three biological replicates of the inner most leaf sheath tissue of S1139E- and S1139RC were harvested after 56 days in the growth chamber. For isolation of RNA, the fungus was grown in potato dextrose broth for 9 days on a shaker (175 rpm) at room temperature.

For RNA isolation, each 1 g sample was ground to a fine powder with liquid nitrogen and resuspended in 10 ml Tri-Reagent (Sigma-Aldrich, St. Louis, Mo, USA). Debris was removed by centrifugation and supernatant was extracted twice with chloroform. RNA in the aqueous layer was precipitated with isopropanol, and the RNA pellet was washed once with ethanol and dissolved in water.

Fungal genomic DNA was extracted from a culture grown in potato dextrose broth for 14 days. The DNA was isolated as previously described [108].

### 454/GS FLX Titanium Sequencing

To provide homologous reference sequence datasets for the SOLiD-SAGE analysis we generated transcriptome sequences for single replicates of plant S1139RC and for the Rose City fungal isolate grown in culture by using the Roche 454 pyrosequencing platform. The endophyte-infected plant was chosen for the 454 sequencing since it was a possibility that some plant and/or fungal genes may only be expressed in endophyte-infected tissue. The longer sequences generated by 454 pyrosequencing facilitated gene identification of the shorter 27 bp SOLiD-SAGE tags. To maximize the transcriptome coverage, the cDNA samples prepared for 454 pyrosequencing were normalized prior to sequencing. cDNA normalization results in an equalization of transcript concentrations in the population so that highly abundant transcripts do not overwelm the subsequent sequencing. In the normalization process the cDNA is denatured and then reassociated. Duplex-specific nuclease then is used to degrade the double-stranded cDNA fraction formed by abundant transcripts. Our samples for 454 pyrosequencing were normalized to obtain sequences for as many different transcripts as possible to aid in the identification of the quantitative SOLiD-SAGE data.

Barcoded cDNA library construction and 454 pyrosequencing were performed by the University of Georgia Genomics Facility. The Evrogen MINT-Universal cDNA synthesis kit (Axxora, LLC, San Diego, CA) was used with a modified oligo(dT) primer.

(5′-AAGCAGTGGTATCAACGCAGAGTAC(T)_4_G(T)_9_C(T)_10_VN-3′) for the first strand synthesis. The Evrogen Trimmer kit (Axxora, LLC) was used to normalize the cDNAs. The cDNA samples were prepared for sequencing by following the rapid library preparation method as described in the GS FLX Titanium manual (454 Life Science, Branford, CT). Further processing was done according to the manufacturer’s protocols. The cDNAs were combined in a ratio of 3∶1 (plant S1139RC:endophyte) and run on 1/4 plate for sequencing. The 454 sequences are available in the GenBank Sequence Read Archive (SRA) database under accession number SRA052297.

### *De novo* 454 Transcriptome Assembly

Raw 454 reads shorter than 50 nucleotides were removed. The *F. rubra* endophyte-infected plant and the *E. festucae* fungal endophyte 454 reads were each assembled without references into contigs by using SeqMan NGen v3.1 (DNAStar Inc., Madison, WI). Parameters for both assemblies were minimum match size of 21 nucleotides, minimum match percentage of 85%, mismatch penalty of 20, and gap penalty of 30. A BlastN comparison of all the endophyte-infected *F. rubra* contigs and singletons against the *E. festucae* E2368 whole genome sequence (GenBank accession ADFL00000000) was done to identify those sequences that originated from the endophyte (e-value cutoff of 1e-05).

### SOLiD-SAGE Sequencing

SOLiD-SAGE libraries were prepared from each of the three biological RNA replicates from plants S1139E- and S1139RC. Each library was generated by using a kit (SOLiD SAGE S3100301, Applied Biosystems, Foster City, CA), which was modified to facilitate barcoding of the samples. Instead of adaptor A supplied in the kit, adaptor Abc (5′-GTACGGCCAAGGCGGATGTACGGTACAGCAGCATG-3′) was used. Adapter Abc contains a 4-bp overhang (CATG), which complements the *Nla*III digested ds cDNA, an *Eco*P15I restriction enzyme recognition site at the 3′ end, and a PCR priming site. Oligos for barcode addition were obtained from the SOLiD Small RNA Expression Kit (Applied Biosystems) and were added to the tags by PCR. Barcode addition, emulsion PCR, and SOLiD sequencing of the libraries were performed at the Waksman Institute Genomics Core Facility, Rutgers University, New Brunswick, NJ. The barcoded libraries were combined and sequenced on one-quarter of a slide on an Applied Biosystems SOLiD 4 System.

### Computational Analysis of SOLiD-SAGE Tags

The characteristics of each SAGE library were determined by using the Galaxy bioinformatics suite (http://main.g2.bx.psu.edu) [109], [110], [111]. The total number of SAGE tags with the *Nla*III restriction enzyme recognition site in each library were first determined. Those SAGE tags containing the *Nla*III site were then analyzed for tags containing homopolymer A (10 or more nucleotides long) at the 3′ end, which is suggestive of SAGE tags containing poly(A)^+^ tails. The total number of putative poly(A)^+^-containing SAGE tags was subtracted from the number of SAGE tags containing *Nla*III site in order to determine the number of potential mappable SAGE tags.

Analysis of the SOLiD-SAGE tags was done by using the Applied Biosystems software program (SOLiD-SAGE v1.10) that maps the SAGE tags to a user supplied sequence database and returns the number of times a particular tag is found in each library. For mapping, the tag length was set to 27 bases and the maximum mismatches allowed was set to two. To separate the plant and fungal SAGE tags, they were mapped in two steps to a reference data set consisting of 1) the S1139RC 454 sequences generated in this study and the *Festuca* and *Lolium* sequences (141,259) downloaded from NCBI or 2) the *E. festucae* 454 sequences generated in this study, *Epichloë* sequences (57,687) downloaded from NCBI, and the whole genome sequence of *E. festucae* isolate E2368 (GenBank accession ADFL02000000).

To identify those plant tags that originated from differentially expressed transcripts, the raw tag counts were normalized by converting to number of tags per million mapped tags in that library. Statistical significance (*P*<0.05) of differential gene expression between plants S1139E- and S1139RC was determined from unpaired t tests by using the PRISM 4 program (GraphPad Software, San Diego, CA). For comparing transcript abundance of the fungal transcripts, the raw tag counts in the replicate libraries were converted to percent of mapped tags.

Gene identification of the plant and fungal SAGE tags was by blast searches of the corresponding 454 sequences to NCBI databases or to the annotated *E. festucae* genome sequence (http://www.endophyte.uky.edu/). The program TargetP (http://www.cbs.dtu.dk/services/TargetP/) [112] was used to predict secreted proteins.

### GO Annotation

GO Slim categorization of the differentially expressed plant transcripts was done by using Blast2GO v.2.5.0 [113], [114], [115], [116] and QuickGO [117; http://www.ebi.ac.uk/QuickGO/GMultiTerm].

### RT-PCR

Oligonucleotide primer sequences used for cDNA synthesis and PCR amplification are presented in Table 6. First-strand cDNA of 4 µg S1139RC total RNA was synthesized from either 500 ng of oligo(dT)_18_ primer or 2 picomoles of a strand-specific primer by using SuperScript™ III Reverse Transcriptase (Life Technologies, Carlsbad, CA) according to the manufacturer’s instructions.

**Table 6:** Sequences of oligonucleotide primers used in this study.

Reference accession	Forward primer, 5′ –3′	Reverse primer, 5′ –3′	Amplicon size (bp)
SRR493691.12929	**Amplification of ** ***Ef-AFP***		
	ATGCAAATCACCGTGGTCGC	CTAATGACACGTGACAGCTC	423 (genomic)279 (cDNA)
ADFL02000476	**Amplification of ** ***Ef-AFP*** ** alternatively spliced variant**		
	AATACCAGACAAAAGGGTCGC	TCAGTCTAGTTCTTCCCTAGA	195 (genomic)195 (cDNA)
SRR493691.19548	**Strand specific amplification of ** ***Nc12*** ** sense transcript**		
	ATTCGCTGGAGAAGACCATG	TAGTCTGGCTAGCAAGAAGG	174
	**Strand specific amplification of ** ***Nc12*** ** antisense transcript**		
	ATGGTCTTCTCCAGCGAATG	CCTGTTACATACTCGCTCTC	192
SRR493691.12929	**Strand specific amplification of ** ***Ef-AFP*** ** sense transcript**		
	GACAATCTGATTCCTCTCTTTC	GACGGGCACTTGACGAACG	250
	**Strand specific amplification of ** ***Ef-AFP*** ** antisense transcript**		
	GTACTTGCATTCGTTTTTGGCT	ACCGTGGTCGCGGTTTTCC	150
SRR493691.55163	**Strand specific amplification of ** ***E. festucae*** ** protease sense transcript**		
	TGGGCGCCGACGGGCAG	TAAATAACCTATACTATTCTCTATA	250
	**Strand specific amplification of ** ***E. festucae*** ** protease antisense transcript**		
	ACATAACAACAGCAACAGACTT	GGCGCCGACGGGCAGAA	150
HO060295.1	**Strand specific amplification of ** ***F. rubra*** ** metallothionein sense transcript**		
	GCGGGAAGATGTACCCTGA	GATGAAGCAATTACAGAGATATA	350
	**Strand specific amplification of ** ***F. rubra*** ** metallothionein antisense transcript**		
	GTCAAACCTGTTTTTCTTAAGTA	GTTTACTTGCTCGCTATGCTA	150

PCR was performed in 20 µL with either 1 µg of fungal genomic DNA, 5 µL *E. festucae*-infected plant cDNA generated from oligo(dT) or 1 µL *E. festucae*-infected plant cDNA generated from a gene-specific primer as templates, 0.25 mM each dNTP, 20 picomoles of each forward and reverse primer (Integrated DNA Technologies, Inc., Coralville, IA), 2 µL of 10X AmpliTaq Buffer and 0.2 µL AmpliTaq Gold DNA polymerase (Life Technologies, Inc., Carlsbad, CA). PCR was done in a GeneAmp 9700 thermocycler (Applied Biosystems, Inc., Foster City, CA). The initial denaturation was conducted at 94°C for 2 min, followed by 35 cycles of 30 s denaturation at 94°C, 30 s annealing at 55°C, and 1 min extension at 72°C, followed by a final extension at 72°C for 10 min. The amplification products were visualized on a 2% agarose gel.

### DNA Sequencing of *Ef-AFP* Splice Variant

Forward and reverse primers were designed based on the *E. festucae* genome sequence (http://www.endophyte.uky.edu/) to amplify the entire region of the *Ef-AFP* gene, and a partial area of the *Ef-AFP* alternatively spliced variant (Table 6). The RT-PCR product of the partial *Ef-AFP* alternatively spliced variant was sequenced directly (Genewiz, Inc., South Plainfield, NJ). For each sequencing reaction, a 10 µl aliquot of the PCR product was treated with 2 µl ExoSAP-IT (USB Corp., Cleveland, OH) to remove unincorporated primers and excess dNTPs. The ExoSAP-IT reaction was performed at 37°C for 15 min followed by heating at 80°C for 15 min to inactivate the enzymes. Sequencing was done in both directions.

### Phylogenetic Analysis

The CLUSTAL-X program [118] was used to align the DNA sequences. For the phylogenetic analyses the sequences were trimmed to include only the regions of sequence overlap for all the sequences in the analysis. The alignments generated by Clustal X were modified manually to minimize gaps. The phylogenetic analyses were performed with the PAUP* program, version 4.0b10 for Macintosh [119]. For both the MCM7 and antifungal protein gene phylogenetic analyses, introns were removed and only the protein coding sequences were used.

The MCM7 phylogenetic analysis was done by using the maximum parsimony full heuristic search option set to random sequence addition, tree-bisection-reconnection (TBR) branch swapping, and Multrees on, with 1,000 bootstrap replications. Gaps were treated as missing data. The *Tuber melanosporum* (Class Pezizomycetes) sequence was designated as the outgroup to root the tree since it is basal to the other species in the tree [120].

The antifungal protein gene phylogenetic tree was done by using an exhaustive maximum parsimony analysis, which returned a single most parsimonious tree. The tree was midpoint rooted.

## Supporting Information

Table S1 A. Plant up-regulated tags; B. Plant down-regulated tags; C. Most abundant endophyte tags
(XLS)
